# Effects of L-/N-Type Calcium Channel Blockers on Angiotensin II–Renin Feedback in Hypertensive Patients

**DOI:** 10.1155/2020/6653851

**Published:** 2020-12-22

**Authors:** Yutaka Kawabata, Takeshi Soeki, Hiroyuki Ito, Tomomi Matsuura, Kenya Kusunose, Takayuki Ise, Koji Yamaguchi, Takeshi Tobiume, Shusuke Yagi, Daiju Fukuda, Hirotsugu Yamada, Tetsuzo Wakatsuki, Mitsuhiro Kitani, Kazuhiro Kawano, Yoshio Taketani, Masataka Sata

**Affiliations:** ^1^Department of Cardiovascular Medicine, Tokushima University Graduate School of Biomedical Sciences, Tokushima, Japan; ^2^Department of Cardio-Diabetes Medicine, Tokushima University Graduate School of Biomedical Sciences, Tokushima, Japan; ^3^Department of Community Medicine for Cardiology, Tokushima University Graduate School of Biomedical Sciences, Tokushima, Japan; ^4^Department of Cardiovascular Medicine, Kagawa Prefectural Shirotori Hospital, Higashikagawa, Japan; ^5^Department of Cardiovascular Medicine, Yoshinogawa Medical Center, Yoshinogawa, Japan; ^6^Department of Cardiovascular Medicine, Shikoku Medical Center for Children and Adults, Zentsuji, Japan

## Abstract

**Objectives:**

Cilnidipine, an L-/N-type calcium channel blocker (CCB), has unique organ-protective properties due to suppression of hyperactivity in the sympathetic nervous system and renin-angiotensin system (RAS). In this study, we hypothesized that cilnidipine might exert a renoprotective effect by suppressing the RAS.

**Methods:**

A total of 25 hypertensive patients receiving a RAS inhibitor were randomly assigned to a cilnidipine (*n* = 12) or amlodipine (*n* = 13) group. The effects of cilnidipine on proteinuria and angiotensin II–renin feedback were assessed.

**Results:**

After 6 months of treatment, both systolic and diastolic blood pressures were significantly reduced to a similar extent in both groups. The urine albumin-to-creatinine ratio was significantly lower in the cilnidipine group (*p* < 0.05) than in the amlodipine group. Amlodipine increased plasma angiotensin I and angiotensin II levels (*p* < 0.05), whereas cilnidipine did not. Interestingly, the cilnidipine group had a higher ratio of angiotensin-(1–7) (Ang-(1–7)) to angiotensin II in plasma than the amlodipine group (*p* < 0.05).

**Conclusions:**

The L-/N-type CCB cilnidipine, but not amlodipine, decreased urinary albumin excretion in hypertensive patients. Cilnidipine also increased the ratio of Ang-(1–7) to angiotensin II in plasma, which might be one factor underlying its beneficial effects.

## 1. Introduction

Patients with chronic kidney disease have been shown to have a higher risk of developing cardiovascular diseases and end-stage renal failure as their blood pressure increases [1]. Therefore, it is crucial to adequately control their blood pressure, initially either with angiotensin-converting enzyme inhibitors or angiotensin II (Ang II) type 1 receptor blockers (ARBs). However, in clinical practice, these drugs are often used in combination with a calcium channel blocker (CCB) to achieve a sufficient antihypertensive effect. There are several types of CCBs that differ according to their duration of action and the calcium channel subtype that they inhibit. The major CCBs exert a strong antihypertensive effect through vasodilation mediated by L-type Ca inhibition, but they can cause reflex stimulation of the sympathetic nervous system and renin-angiotensin system (RAS) by their abrupt hypotensive action. Cilnidipine, a dihydropyridine CCB, is an antihypertensive drug with both L-type and N-type calcium channel antagonist activities [2, 3]. Since cilnidipine suppresses hyperactivity of the sympathetic nervous system, it exerts a slow and sustained antihypertensive effect without causing reflex tachycardia during hypotension.

Previous studies showed that cilnidipine demonstrates unique organ-protective properties as a result of its effects on the sympathetic nervous system [4–6]. In addition, we reported that cilnidipine exerts a greater renoprotective effect than amlodipine due to its antioxidative properties [7]. Furthermore, previous studies demonstrated that cilnidipine is unique in that it suppresses the RAS. Some previous studies reported that cilnidipine has no effect on plasma renin activity or plasma Ang II levels, while amlodipine, an L-type CCB, significantly increases both parameters [8, 9]. Another study showed that cilnidipine but not amlodipine, suppressed the ARB-induced increase in plasma renin activity and plasma Ang II levels [10].

Angiotensin-(1–7) (Ang-(1–7)), a heptapeptide member of the RAS, has attracted attention in recent studies as an antagonist of Ang II. Ang-(1–7) binds to Mas receptors, seven transmembrane proteins also known as the G-protein-coupled receptors via different signaling pathways. Ang-(1–7) exerts antiproliferative, antigrowth, and cardiorenal protective effects in vascular smooth muscle cells, cardiac myocytes, and fibroblasts. These actions contrast with the Ang II-mediated proliferative effects on smooth muscle cells and fibroblasts [11–13]. Ang-(1–7) is formed by two pathways. In one pathway, Ang I is converted to Ang II by angiotensin-converting enzyme (ACE), and then Ang II is converted to Ang-(1–7) by Ang II-converting enzyme (ACE2). In the other pathway, Ang I is converted to angiotensin-(1–9) (Ang-(1–9)) by ACE2, and then Ang-(1–9) is converted to Ang-(1–7) by ACE [14]. However, the precise mechanism of the renoprotective effect of cilnidipine via Ang-(1–7) remains unknown in hypertensive patients. Therefore, in this study, we hypothesized that cilnidipine might exert a renoprotective effect by the activation of the ACE2-Ang-(1–7) pathway and suppression of the ACE-Ang II–angiotensin II type 1 receptor (AT1R) pathway.

## 2. Methods

### 2.1. Study Design

In this 6-month, multicenter, prospective, randomized, open-label clinical trial named the Tokushima Antioxidation Clinical Trial in Hypertensives, Cilnidipine vs. Amlodipine (TACTICAL2) study (UMIN ID: 00006544), 25 hypertensive patients receiving ARB treatment at four hospitals in Japan between August 2013 and June 2016 were enrolled. The study was approved by the Institutional Review Board of The University of Tokushima Clinical Research Center and the review boards of the four hospitals. The study protocol was in accordance with the Declaration of Helsinki. Written informed consent was obtained from all patients included in the study. The enrollment criteria were as follows: (1) hypertension defined as a blood pressure (BP) of ≥140/90 mmHg and (2) treatment with ARBs for 3 months or more before the administration of cilnidipine or amlodipine. The exclusion criteria were as follows: (1) age <45 years or >85 years, (2) hypertensive emergency, (3) severe liver failure, (4) pregnancy, and (5) a history of severe adverse effects due to CCBs or ARBs.

**Figure 1:**
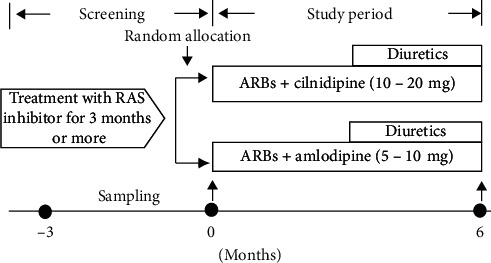
Study design. Patients received either 10 mg per day of cilnidipine, which was increased to a daily dose of 20 mg, or 5 mg per day of amlodipine, which was increased to a daily dose of 10 mg. Doses of RAS inhibitors were not altered when the combination with amlodipine failed to reduce BP to the target level within 3 months. Instead, additional antihypertensive medications, such as diuretics, were administered to achieve the target BP.

The overview of the study design is shown in Figure 1. Subjects were randomly assigned to two groups at the time of registration. To achieve the target BP level of 130/85 mmHg, patients received either 10 mg per day of cilnidipine, which was titrated upward to a daily dose of 20 mg, or 5 mg per day of amlodipine, which was titrated upward to a daily dose of 10 mg. The doses of ARBs were not altered during the study period. BP was measured according to the Japanese Society of Hypertension Guidelines for the Measurement of Hypertension.

During the first 3 months of the study, patients received their initial (baseline) assigned drug combination. However, if cilnidipine or amlodipine in combination with the ARB failed to reduce BP to the target level within 3 months, additional antihypertensive medications, such as diuretics, were administered until the target was reached.

### 2.2. Measurement of the Urinary Parameters

Morning spot urine samples were obtained. The urinary levels of albumin and creatinine were measured using a turbidimetric immunoassay and enzyme assay, respectively (LSI Medience Corporation, Tokyo, Japan).

### 2.3. Measurement of RAS

The levels of Ang I, Ang II, and Ang-(1–7) concentrations in human plasma were measured by liquid chromatography (LC)/mass spectrometry (MS)/MS (SRL, Tokyo, Japan).

### 2.4. Statistical Analysis

The data in this study are expressed as the mean ± SD. Differences between the two groups were analyzed by Student's unpaired *t*-test for continuous variables and the chi-square test for categorical variables. Changes before and after treatment administration were assessed by Wilcoxon's signed-rank test.

## 3. Results

### 3.1. Baseline Characteristics

The baseline characteristics of the subjects are shown in Table 1. There were no significant differences between the two groups in terms of age, sex, systolic and diastolic BP, heart rate, HbA1c, or total cholesterol level. There was no additional use of diuretics in either group during the study period.

### 3.2. Safety

No adverse effects related to the antihypertensive treatments occurred during the study period.

### 3.3. Changes in BP and Heart Rate

**Figure 2:**
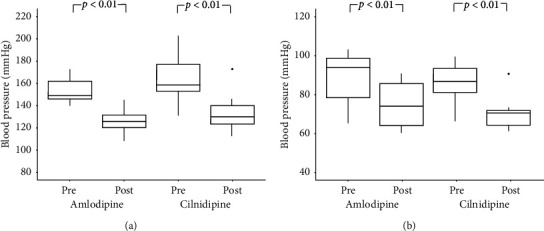
Changes in BP during the study period. BP was similar between the cilnidipine and amlodipine groups. The systolic and diastolic BP values were significantly reduced in both groups after 6 months of treatment. BP, blood pressure. (a) Systolic blood pressure. (b) diastolic blood pressure.

**Figure 3:**
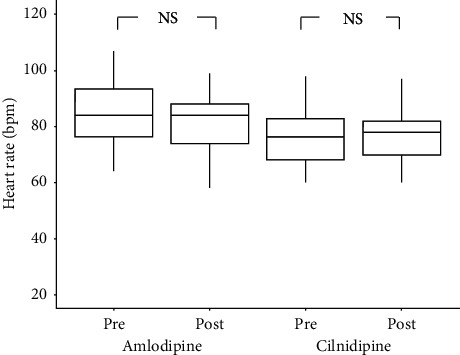
Changes in heart rate during the study period. Heart rate was almost the same between the cilnidipine and amlodipine groups and remained unchanged after 6 months of treatment.

The systolic and diastolic BP values were significantly lower in both the cilnidipine and amlodipine groups at 6 months after the initiation of therapy than at baseline (*p* < 0.01). However, there were no significant differences in BP values between the two groups at either time point (shown in Figure 2). Moreover, the heart rate showed no changes over time in either group (shown in Figure 3).

### 3.4. Changes in Urinary Albumin

**Figure 4:**
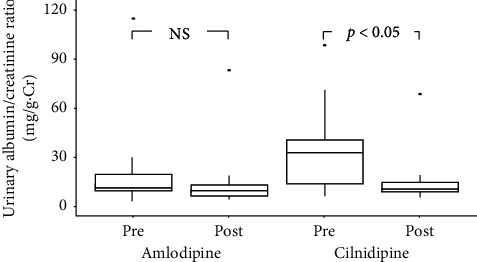
Changes in the urine albumin-to-creatinine ratio before and after the treatment. After 6 months of treatment, the urine albumin-to-creatinine ratio was suppressed in the cilnidipine group but not the amlodipine group.

While the urine albumin-to-creatinine ratio did not change over time in the amlodipine group, it decreased significantly after 6 months of treatment in the cilnidipine group (*p* < 0.05; shown in Figure 4).

### 3.5. Changes in Plasma Ang I and Ang II Levels

**Figure 5:**
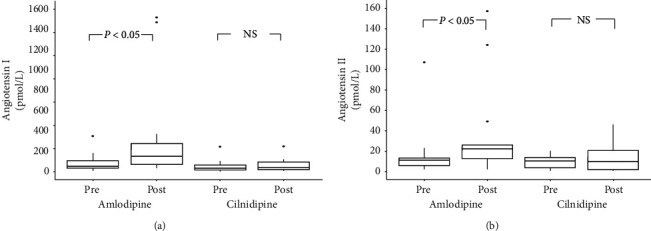
Changes in the plasma Ang I (a) and Ang II (b) levels before and after the treatment. After 6 months of treatment, significant increases in both plasma Ang I and Ang II levels were observed in the amlodipine group but not in the cilnidipine group. Ang I, angiotensin I; Ang II, angiotensin II.

After 6 months of treatment, the plasma Ang I and Ang II levels were significantly increased in the amlodipine group (*p* < 0.05) but not in the cilnidipine group (shown in Figure 5).

### 3.6. Plasma Aldosterone Levels and Plasma Renin Activity (PRA)

**Figure 6:**
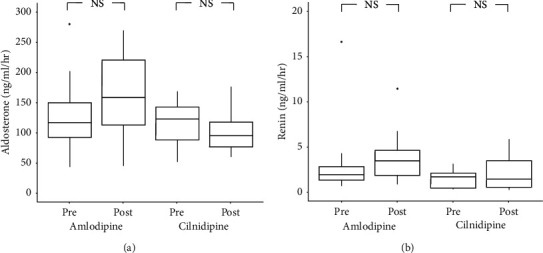
Plasma aldosterone levels and PRA before and after treatment. Although there were no significant differences in plasma aldosterone levels of PRA between the 2 groups, both showed nonsignificant increases in the amlodipine group but not in the cilnidipine group. PRA, plasma renin activity.

Although neither plasma aldosterone levels nor PRA differed significantly between the two groups, there was a nonsignificant increase in both measurements from pre- to post-treatment in the amlodipine group (shown in Figure 6).

### 3.7. Ratio of Ang-(1–7) to Ang II in Plasma

**Figure 7:**
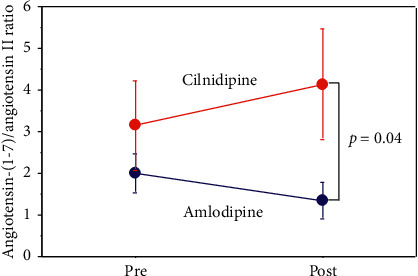
Changes in the ratio of Ang-(1–7) to Ang II in plasma before and after treatment. After 6 months of treatment, the cilnidipine group had a significantly higher ratio Ang-(1–7) to Ang II in the plasma than the amlodipine group. Ang-(1–7), angiotensin; Ang II, angiotensin II.

Compared to the amlodipine group, the cilnidipine group had a significantly higher ratio of Ang-(1–7) to Ang II in plasma at the end of treatment (*p* < 0.05; shown in Figure 7).

## 4. Discussion

In this study, we evaluated whether cilnidipine might exert a renoprotective effect by suppressing the RAS. The results suggested that cilnidipine, unlike amlodipine, had a greater suppressive effect on the RAS when used in combination with a RAS inhibitor to treat hypertensive patients. In addition, the finding that cilnidipine had greater antiproteinuric effects than amlodipine was consistent with our previous study.

In this study, amlodipine increased both plasma Ang I and Ang II levels (*p* < 0.05), whereas cilnidipine did not. Furthermore, cilnidipine raised the ratio of Ang-(1–7) to Ang II in plasma more than amlodipine did. These results suggest that cilnidipine might shift the balance of the 2 arms of the RAS from the ACE-Ang II-AT1R receptor to the ACE2-Ang-(1–7)-Mas receptor. According to previous studies, the predominant effects of the RAS might be related to the balance between these 2 arms [13–15]. This effect of cilnidipine may be partly caused by N-type calcium channel blockade via the close conjunction between the ACE2-Ang-(1–7) pathway and sympathetic nerve activity [16]. It is still unknown if Ang-(1–7) is predominantly formed from Ang II via ACE2 or from Ang-(1–9) via ACE. However, despite the similar hypotensive effects of cilnidipine and amlodipine when combined with RAS inhibitor therapy, the greater increase in the ratio of Ang-(1–7) to Ang II by cilnidipine might contribute to its superior renal and cardiovascular protective effects. This hypothesis is supported by previous reports showing that Ang-(1–7) administration to streptozotocin (STZ)-induced diabetic rats reduced proteinuria and restored vascular reactivity in isolated renal artery segments [17] and that treatment with Ang-(1–7) attenuated NAPDH oxidase activation and reduced proteinuria in spontaneously hypertensive, STZ-induced diabetic rats [18].

We previously showed that in hypertensive patients, cilnidipine decreased urinary levels of both 8-hydroxy-20-deoxyguanosine (OHdG), a biomarker of oxidative stress, and liver-type fatty-acid–binding protein (L-FABP), a useful biomarker for renal tubulointerstitial injury [7]. Cilnidipine may suppress oxidative stress by modulating the renal RAS system. In practice, the increased ratio of Ang-(1–7) to Ang II might have attenuated the significant oxidative stress that contributed to renal injury in this study, because previous reports have shown that Ang-(1–7) has an antioxidative effect in vascular tissues whereas Ang II has an oxidative effect [19, 20]. In agreement with these data, other studies have shown that Ang-(1–7) mediates the effects of ACE2 in suppressing Ang II–induced oxidative stress and ERK1/2 signaling in cultured cardiomyocytes and fibroblasts [21] and that ACE2 attenuates cardiac, kidney, and vascular oxidative stress in SHR [22].

We previously reported that cilnidipine, but not amlodipine, reduced urinary albumin excretion in hypertensive patients receiving RAS inhibitor treatment [7]. In addition, the Cilnidipine vs. Amlodipine Randomized Trial for Evaluation in Renal Disease (CARTER) study showed that cilnidipine was more beneficial than amlodipine in hypertensive patients [23]. Supporting these previous reports, this study showed that only cilnidipine reduced urinary albumin excretion in hypertensive patients receiving ARB treatment. A recent study reported that L/N-type CCBs exert the antialbuminuric and renoprotective effects at least in part by reducing glomerular hypertension through both efferent and afferent arteriolar vasodilation [24]. Furthermore, another study reported that in patients with diabetes, the combination of cilnidipine with a RAS inhibitor had an additive effect in suppressing urinary albumin excretion [25].

Although the present study revealed the association between the renoprotective effects of cilnidipine and the systemic RAS, it remains unknown whether cilnidipine also have an effect on the renal ACE2-Ang-(1–7)-Mas receptors which might contribute to the renoprotection. Fan et al. reported the presence of N-type Ca channels in cultured rat glomerular epithelial cells [26]. In addition, they showed that treatment with cilnidipine, but not amlodipine, significantly suppressed the renal Ang II levels and podocyte injury in spontaneously hypertensive rat/ND mcr-cp, suggesting that the renoprotective mechanism of cilnidipine may involve the suppression of RAS. Furthermore, Toba et al. showed that cilnidipine inhibited the renal renin-angiotensin-aldosterone system, including measurements of renal ACE activity and aldosterone content in the DOCA-salt hypertensive rats [27]. Further studies are needed to clarify the association between the renoprotective effects of cilnidipine and the local RAS including the renal ACE2-Ang-(1–7)-Mas receptors in the kidney.

### 4.1. Limitations

Our study had several limitations. First, serial measurements of urinary and RAS parameters could only be performed in a limited number of patients at 2 time points. Second, measurements related to the RAS might have fluctuated over the time and position of the patient during the blood sampling. Third, the failure to consider the severity of chronic kidney disease may have influenced the results of the study. Fourth, we did not clarify the precise mechanism whereby cilnidipine suppresses the RAS and oxidative stress. Finally, since the sample size was small in this study, we need to increase the number of samples in future evaluations.

## 5. Conclusion

In conclusion, cilnidipine and ARB combination therapy reduced urinary albumin excretion and increased the ratio of Ang-(1–7) to Ang II in plasma in hypertensive patients. Better renoprotective effects may be achieved by combining a RAS inhibitor with an L-/N-type CCB rather than an L-type CCB.

## Figures and Tables

**Table 1:** Characteristics of the study patients.

	Cilnidipine group (*n* = 12)	Amlodipine group (*n* = 13)	*p* value
Age (years)	68.5 ± 8.8	62.9 ± 11.1	NS
Sex (male/female)	6/6	5/8	NS
Systolic BP (mmHg)	162.1 ± 19.4	149.9 ± 18.7	NS
Diastolic BP (mmHg)	86.2 ± 9.8	88.6 ± 12.8	NS
Heart rate (bpm)	76.6 ± 11.6	84.8 ± 12.7	NS
HbA1c (%)	6.0 ± 0.4	6.2 ± 0.8	NS
Total cholesterol (mg/dL)	194.0 ± 43.8	194.9 ± 31.6	NS

Values are the mean ± standard deviation.

## Data Availability

The data used to support the findings of this study are available from the corresponding author upon request.
